# Synthesis of New Glycosylated Flavonoids with Inhibitory Activity on Cell Growth

**DOI:** 10.3390/molecules23051093

**Published:** 2018-05-05

**Authors:** Ana R. Neves, Marta Correia-da-Silva, Patrícia M. A. Silva, Diana Ribeiro, Emília Sousa, Hassan Bousbaa, Madalena Pinto

**Affiliations:** 1 Laboratory of Organic and Pharmaceutical Chemistry, Department of Chemical Sciences, Faculty of Pharmacy, University of Porto, Rua Jorge Viterbo Ferreira, 228, 4050-313 Porto, Portugal; anarcneves92@gmail.com (A.R.N.); m_correiadasilva@ff.up.pt (M.C.-d.-S.); madalena@ff.up.pt (M.P.); 2 Interdisciplinary Centre of Marine and Environmental Research (CIIMAR), University of Porto, Terminal de Cruzeiros do Porto de Leixões, Avenida General Norton de Matos, S/N 4450-208 Matosinhos, Portugal; hassan.bousbaa@iucs.cespu.pt; 3 CESPU, Institute of Research and Advanced Training in Health Sciences and Technologies (IINFACTS), Rua Central de Gandra, 1317, 4585-116 Gandra, Portugal; patricia_masilva@hotmail.com (P.M.A.S.); dianaroberta@ua.pt (D.R.)

**Keywords:** flavonoids, xanthones, growth inhibitory activity, acetylation, glycosylation

## Abstract

Natural flavonoids and xanthone glycosides display several biological activities, with the glycoside moiety playing an important role in the mechanism of action of these metabolites. Herein, to give further insights into the inhibitory activity on cell growth of these classes of compounds, the synthesis of four flavonoids (**5**, **6**, **9**, and **10**) and one xanthone (**7**) containing one or more acetoglycoside moieties was carried out. Acetyl groups were introduced using acetic anhydride and microwave irradiation. The introduction of one or two acetoglycoside moieties in the framework of 3,7-dihydroxyflavone (**4**) was performed using two synthetic methods: the Michael reaction and the Koenigs-Knorr reaction. The in vitro cell growth inhibitory activity of compounds **5**, **6**, **7**, **9**, and **10** was investigated in six human tumor cell lines: A375-C5 (malignant melanoma IL-1 insensitive), MCF-7 (breast adenocarcinoma), NCI-H460 (non-small cell lung cancer), U251 (glioblastoma astrocytoma), U373 (glioblastoma astrocytoma), and U87MG (glioblastoma astrocytoma). The new flavonoid 3-hydroxy-7-(2,3,4,6-tetra-*O*-acetyl-β-glucopyranosyl) flavone (**10**) was the most potent compound in all tumor cell lines tested, with GI_50_ values < 8 μM and a notable degree of selectivity for cancer cells.

## 1. Introduction

**Figure 1:**
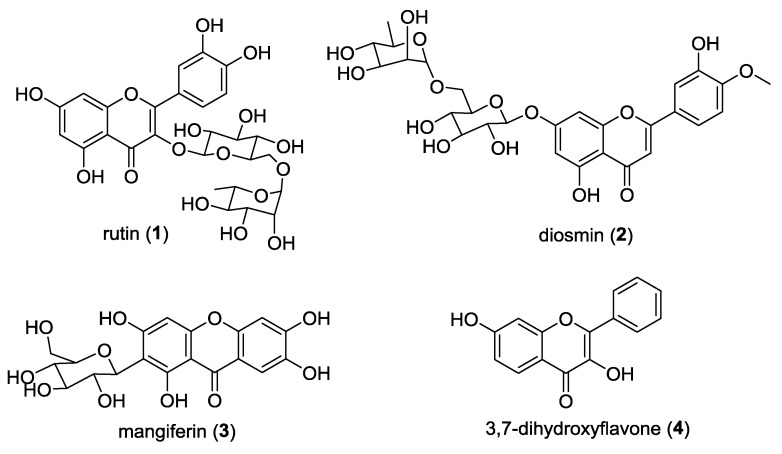
Structure of the selected precursors **1**–**4**.

Flavonoid and xanthone derivatives comprise two classes of oxygenated heterocycles associated with a large number of biological and pharmacological activities [1,2]. These compounds also occur in nature as glycosides, which have been shown to have several biological activities, namely, antimicrobial [3,4,5], antioxidant [6,7,8,9], and antitumor [10,11]. Flavonoid glycosides are widely present in vegetables and fruits and can be found with different types of glycosides, while xanthone glycosides are mainly found in the families Gentianaceae and Polygalaceae with a glucose moiety [12]. In particular, acetylated flavonoid and xanthone glycosides have been reported as bioactive compounds with antioxidant [13,14] and antitumor activities [15,16,17,18,19]. The last step in flavonoid biosynthesis is, in some plant species, terminated by acylation, which is known to increase the solubility and stability of glycosylated flavonoids in lipophilic systems [20]. Thus, we were inspired to perform acetylation and glycosylation in natural flavonoids and xanthones. Rutin (**1**), diosmin (**2**), and mangiferin (**3**) were selected for acetylation due to their use in therapy and/or investigation in clinical trials, and 3,4-dihydroxyflavone (**4**) was selected for glycosylation to study the structure-activity relationship (Figure 1). All these starting materials have previously been shown to display in vitro and in vivo antitumor activity [11,21,22,23,24].

## 2. Results and Discussion

### 2.1. Synthesis

**Scheme 1:**
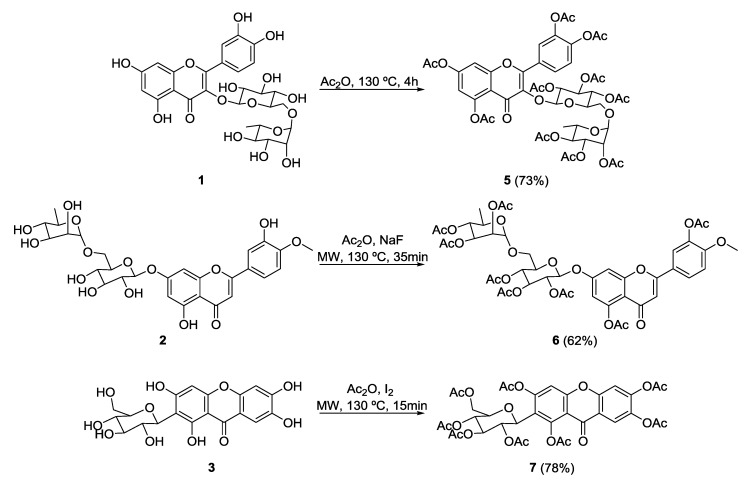
Synthesis of rutin peracetate (**5**), diosmin peracetate (**6**), and mangiferin peracetate (**7**). Ac_2_O—Acetic anhydride; MW—Microwave.

Rutin peracetate (**5**) was obtained from rutin (**1**) according to a classical acetylation method using acetic anhydride under reflux for 4 h in solvent-free conditions (Scheme 1) [25]. Previously, compound **5** was obtained in the presence of pyridine with long reaction times [26]. The synthesis of compound **5** without using pyridine and with short reaction times is reported for the first time.

Several products that were difficult to separate were obtained when this acetylation method was applied to diosmin (**2**) and mangiferin (**3**). Diosmin peracetate (**6**) was successfully obtained in only 35 min (62% yield) in the presence of NaF, under microwave (MW) irradiation (Scheme 1) [27]. Peracetylation of mangiferin (**3**) was achieved in only 15 min (78% yield) under MW irradiation, but only when iodine was used as a catalyst (Scheme 1).

Previous methods described to synthesize compounds **6** and **7** used highly toxic pyridine as solvent and catalyst, which was applied room temperature, leading to long reaction times [28,29,30,31,32]. Herein, the application of MW irradiation without pyridine allowed the synthesis of compounds **6** and **7** in short reaction times.

**Scheme 2:**
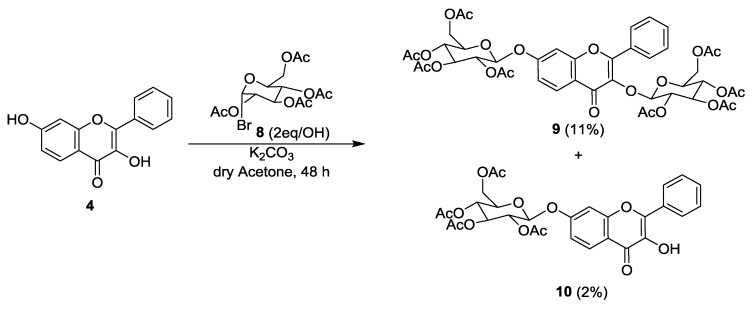
Synthesis of 3,7-(2,3,4,6-tetra-*O*-acetyl-β-glucopyranosyl) flavone (**9**) and 3-hydroxy-7-(2,3,4,6-tetra-*O*-acetyl-β-glucopyranosyl) flavone (**10**).

For glycosylation of flavone **4**, 2,3,4,6-tetra-*O*-acetyl-α-d-glucopyranosyl bromide (**8**) was selected as glucose donor and two reaction methods were applied: a Koenigs-Knorr method [33,34,35] and a modified Michael method [36]. Both reactions were carried out at room temperature to decrease the number of side products [37]. Formation of glycosylated products could not be observed using the Koenigs-Knorr approach, even with the addition of 3 equivalents/OH of 2,3,4,6-tetra-*O*-acetyl-α-d-glucopyranosyl bromide (**8**). Using the modified Michael method, with K_2_CO_3_ in dry acetone, it was possible to obtain two glycosylated products (Scheme 2), the 3,7-(2,3,4,6-tetra-*O*-acetyl-β-glucopyranosyl) flavone (**9**) and the 3-hydroxy-7-(2,3,4,6-tetra-*O*-acetyl-β-glucopyranosyl) flavone (**10**), although with low yields (11% and 2%, respectively).

### 2.2. Structure Elucidation

The structure of compounds **5**–**7** was established comparing their ^1^H and ^13^C NMR data with the reported in the literature [29,30,38,39], while the structures of the compounds **9** and **10** was elucidated for the first time, using infrared (IR), nuclear magnetic resonance (NMR), and high-resolution mass spectrometry (HRMS) techniques (Figures S1–S4).

**Figure 2:**
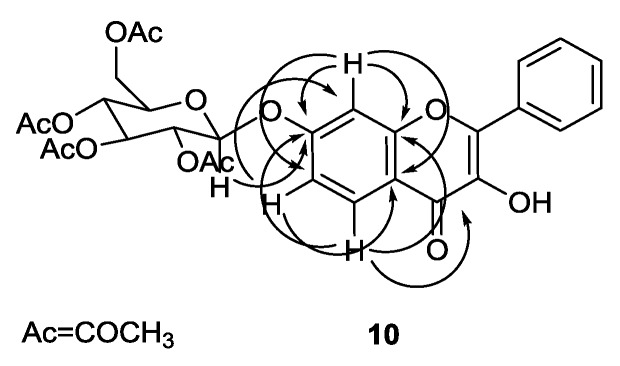
Main connectivities found in the HMBC spectrum of compound **10**.

For instance, the IR spectrum of compound **9** showed a strong band at 1750 cm^−1^, which is characteristic of the C=O ester stretching vibration. ^1^H and ^13^C NMR spectra of compound **9** indicated the presence of a flavone moiety and of two sugar moieties. ^1^H and ^13^C NMR spectra of compound **10** indicated the presence of a flavone moiety and one acetylated sugar moiety. The assignments of the carbon atoms directly bonded to proton atoms were achieved from heteronuclear single quantum correlation (HSQC) experiments, and the chemical shifts of the carbon atoms not directly bonded to proton atoms were deduced from heteronuclear multiple bond correlation (HMBC) correlations. For instance, Figure 2 shows the correlations found for compound **10**.

The position of the sugar moiety in compound **10** was evidenced by the correlation found in the HMBC spectrum between the proton signals of H-1″ (δ_H_ 5.16–5.36 ppm) and the carbon signal of C-7 (δ_C_ 160.9 ppm).

### 2.3. Growth Inhibition Activity in Human Tumor Cell Lines

Acetylated flavonosides **5**, **6**, **9**, **10**, and acetylated xanthonoside **7**, as well as their precursors, compounds **1**–**4**, were evaluated for their in vitro growth inhibitory effect on six human tumor cell lines: A375-C5 (IL-1 insensitive malignant melanoma), MCF-7 (breast adenocarcinoma), NCI-H460 (non-small-cell lung cancer), U251 (glioblastoma astrocytoma), U373 (glioblastoma astrocytoma), and U87MG (glioblastoma astrocytoma). For the tested compounds, a dose–response curve was established, and the results, summarized in Table 1, are expressed as the concentration that was able to cause 50% cell growth inhibition (GI_50_).

Concerning the precursors, flavone **4** was the only precursor that showed an inhibitory effect on the growth of IL-1 insensitive A375-C5, estrogen-dependent MCF-7, and NCI-H460 cell lines.

In contrast to compounds **5**, **7**, and **10**, diosmin peracetate (**6**) did not show an inhibitory effect on the growth of any of the six human tumor cell lines tested. Rutin peracetate (**5**) exhibited a good growth inhibitory activity in all the cell lines tested, mangiferin peracetate (**7**) showed a moderate growth inhibitory activity only in IL-1 insensitive A375-C5, estrogen-dependent MCF-7, and NCI-H460 cell lines, while compound **10** was the most potent compound in all human tumor cell lines tested, with GI_50_ values < 8 μM.

The presence of the acetyl groups in compounds **5** and **7** was found to be crucial for their cell growth inhibitory activity (comparing with compounds **1** and **3**, respectively). Acylation has been a strategy used to increase the penetration of flavonoids through the cell membrane [41,42]. When comparing the peracetylated compounds (**5**–**7**, **9**), some correlations can be found between lipophilicity and the cell growth inhibitory effect, especially in melanoma cells; compound **5**, with the highest calculated logP value, was also associated with the highest inhibitory effect. It is noteworthy that compound **10**, the only investigated acetylated glycoside with a free hydroxyl, was the most active derivative, highlighting the importance of both types of substituents. The position of the sugar moiety in flavonoids seems to be critical for the inhibitory activity.

The selectivity index was calculated for compounds **4**, **5**, **7**, **9**, **10**, and for Doxorubicin (Table 2).

As outlined in Table 2, the results clearly reflect a notable degree of selectivity of compound **10** in all cell lines studied. Of note, human breast cancer cell line MCF-7 responded very well by exhibiting a higher selectivity index than the other cancer cell lines. As to the other compounds, some had no selectivity (**4** and **7**) while the others (**5**) or (**9**) exhibited some selectivity regarding the cancer lines in which they were most active. Interestingly, all tested compounds exhibited a better degree of selectivity than Doxorubicin.

## 3. Materials and Methods

### 3.1. General Information

Commercially available reagents were purchased from Sigma-Aldrich Co. (Sintra, Portugal) and BioSolve (Dieuze, France). Dichloromethane was dried using the following procedure: pre-drying with CaSO_4_ and reflux over calcium hydride and distillation onto 4 Å molecular sieves [43]. Extra-dry acetone was purchased from BioSolve (France). Reactions were controlled by thin layer chromatography (TLC) using Merck silica gel 60 (GF254) plates. Compounds were visually detected by absorbance at 254 and/or 365 nm and ferric chloride 10% in methanol (MeOH). Purification of compounds by flash chromatography was carried out using Fluka silica gel 60 (0.04–0.063 mm). MW reactions were performed using a MicroSYNTH 1600 from Milestone (ThermoUnicam, Lisboa, Portugal) synthesizer in open reaction vessels. IR spectra were obtained in KBr microplate in a FTIR spectrometer Nicolet is 10 from Thermo Scientific with Smart OMNI-Transmission accessory (Software OMNIC 8.3, Thermo Scientific, Madison, WI, USA). ^1^H and ^13^C NMR spectra were performed in University of Aveiro, Department of Chemistry and were taken in CDCl_3_ or DMSO-*d*_6_, at room temperature, on Bruker Avance 300 instrument (300.13 MHz for ^1^H and 75.47 MHz for ^13^C, Bruker Biosciences Corporation, Billerica, MA, USA). ^13^C NMR assignments were made by 2D HSQC and HMBC experiments (long-range C, H coupling constants were optimized to 7 and 1 Hz). Chemical shifts are expressed in ppm values relative to tetramethylsilane (TMS) as an internal reference. Coupling constants are reported in hertz (Hz). HRMS mass spectra were measured on an APEX III mass spectrometer, recorded as ESI (Electrospray) made in Centro de Apoio Científico e Tecnolóxico á Investigation (CACTI, University of Vigo, Spain). Six human tumor cell lines were used to study antitumor activity: MCF-7 ER (+) (breast adenocarcinoma), NCI-H460 (non-small-cell lung cancer), and A375-C5 (melanoma), U251 (glioblastoma astrocytoma), U373 (glioblastoma astrocytoma) and U87MG (glioblastoma astrocytoma).

### 3.2. Chemistry

*Synthesis of rutin peracetate* (**5**)*.* Rutin (**1**, 0.3 g, 0.5 mmol) was added to acetic anhydride (12 mL) and the mixture was heated at 130 °C, for 4 h. The solution obtained was poured into ice and extracted with CH_2_Cl_2_. The organic layer was extracted with a saturated solution of NaHCO_3_, dried with anhydrous Na_2_SO_4_, filtered, and then evaporated under reduced pressure. The obtained oil was dissolved in ethyl acetate. A solid material was obtained by adding petroleum ether 60–80 °C corresponding to 2-(3,4-di-*O*-acetylphenyl)-5,7-di-*O*-acetyl-3-[3,4,5-tri-*O*-acetyl-α-l-rhamnopyranosyl-(1→6)-3,4,5-tri-*O*-acetyl-β-d-glucopyranosyloxy]-4*H*-chromen-4-one (**5**) (0.43 g, 0.41 mmol, 73% yield); mp 119–120 °C (petroleum ether 60–80 °C).

*Synthesis of diosmin peracetate* (**6**). Diosmin (**2**, 0.05 g, 0.08 mmol) and NaF (0.42 g, 10 mmol) were mixed in acetic anhydride (4 mL) and the mixture was kept under MW irradiation (400 W) at 130 °C, for 35 min. After cooling, the solution obtained was poured into ice and then extracted with CH_2_Cl_2_. The organic layer was extracted with NaHCO_3_ followed by crystallization from MeOH/H_2_O provided 5,3-*O*-acetyl-2-(3-*O*-acetyl-4-methoxyphenyl)-7-[2,3,4-tri-*O*-acetyl-α-l-rhamnopyranosyl-(1→6)-2,3,4-tri-*O*-acetyl-β-d-glucopyranosyloxy] oxychromen-4-one (**6**) as a yellow solid (0.048 g, 0.051 mmol, 62% yield); mp 135–138 °C.

*Synthesis of mangiferin peracetate* (**7**)*.* Mangiferin (**3**, 0.2 g, 0.5 mmol) and iodine (0.009 g, 0.07 mmol) were mixed in acetic anhydride (7 mL) and the reaction was kept under MW irradiation (400 W), at 130 °C, for 15 min. After cooling, a saturated solution of sodium thiosulfate was added to convert iodine (dark yellow) into iodide (yellow). The crude product was extracted with CH_2_Cl_2_ and the organic layer was extracted with a saturated solution of NaHCO_3_ twice, dried with anhydrous Na_2_SO_4_, and filtered. The solvent was evaporated under reduced pressure and the oil obtained was dissolved in ethyl acetate. A yellow solid was obtained with petroleum ether 60–80 °C corresponding to 1,3,6,7-tetra-*O*-acetyl-2-*C*-(2,3,4,6-tetra-*O*-acetyl-β-d-glucopyranosyl)-9*H*-xanthen-9-one (**7**) (0.294 g, 0.39 mmol, 78% yield); mp 143–147 °C (petroleum ether 60–80 °C).

*Synthesis of 3,7-(2,3,4,6-tetra-O-acetyl-β-glucopyranosyl) flavone* (**9**) *and 3-hydroxy-7-(2,3,4,6-tetra-O-acetyl-β-glucopyranosyl) flavone* (**10**). 3,7-Dihydroxyflavone (**4**, 0.100 g, 0.4 mmol) and K_2_CO_3_ (0.225 g, 1.57 mmol, 2eq/OH) were mixed in dry acetone (10 mL) and the mixture was kept under stirring for 15 min. 2,3,4,6-Tetra-*O*-acetyl-α-d-glucopyranosyl bromide (**8**, 0.653 g, 1.57 mmol, 2eq/OH) was added and the mixture was kept under stirring at room temperature, for 48 h. The suspension was filtered to eliminate the K_2_CO_3_ and the filtrate was evaporated. The oil obtained was further purified through flash column chromatography (100% CHCl_3_). The obtained fractions were gathered and crystallized from MeOH to achieve 3,7-(2,3,4,6-tetra-*O*-acetyl-β-glucopyranosyl) flavone (**9**, 0.04 g, 0.045 mmol, 11.4% yield) and 3-hydroxy-7-(2,3,4,6-tetra-*O*-acetyl-β-glucopyranosyl) flavone (**10**, 0.003 g, 0.004 mmol, 1.2% yield).

*3,7-(2,3,4,6-Tetra-O-acetyl-β-glucopyranosyl) flavone* (**9**). Mp 105–108 °C; IR (KBr) υ_max_: 2962, 2917, 2843, 1750, 1623, 1449, 1381, 1231, 1066, 1040 cm^−1^; ^1^H NMR (CDCl_3_, 300.13 MHz) δ: 8.17 (1H, d, *J =* 8.8 Hz, H-5), 8.02–7.98 (2H, m, H-2′,6′), 7.50–7.47 (3H, m, H-3′,4′,5′), 7.10 (1H, d, *J =* 2.2 Hz, H-8), 7.05 (1H, dd, *J =* 2.3 and 8.9 Hz, H-6), 5.71 (1H, d, *J =* 7.7 Hz, H-glucose), 5.34–5.04 (7H, m, H-glucose), 4.31–4.19 (2H, m, H-glucose), 3.98–3.91 (3H, m, H-glucose), 3.65–3.60 (1H, m, H-glucose), 2.09 (3H, s, COCH_3_), 2.08 (3H, s, COCH_3_), 2.06 (3H, s, COCH_3_), 2.05 (3H, s, COCH_3_), 2.04 (3H, s, COCH_3_), 2.01 (3H, s, COCH_3_), 2.00 (3H, s, COCH_3_), 1.90 (3H, s, COCH_3_) ppm; ^13^C NMR (CDCl_3_, 75.47 MHz) *δ*: 173.4 (C-4), 170.6 (COCH_3_), 170.5 (COCH_3_), 170.3 (COCH_3_), 170.2 (COCH_3_), 170.0 (COCH_3_), 169.7 (COCH_3_), 169.5 (COCH_3_), 169.4 (COCH_3_), 160.9 (C-7), 157.4 (C-2), 156.6 (C-9), 136.3 (C-3), 131.0 (C-1′), 130.6 (C-4′), 129.1 (C-3′,5′), 128.3 (C-2′,6′), 127.6 (C-5), 119.9 (C-10), 115.5 (C-6), 104.4 (C-8), 98.9 (C-glucose), 98.5 (C-glucose), 72.9 (C-glucose), 72.6 (C-glucose), 72.6 (C-glucose), 71.8 (C-glucose), 71.8 (C-glucose), 68.4 (C-glucose), 68.2 (C-glucose), 62.1 (C-glucose), 61.5 (C-glucose), 21.0 (COCH_3_), 20.8 (COCH_3_), 20.8 (COCH_3_), 20.7 (COCH_3_), 20.7 (COCH_3_) ppm; HRMS (ESI^+^) *m*/*z* calcd for C_29_H_28_O_13_ [M + H] 915.25535, found 915.25410.

*3-Hydroxy-7-(2,3,4,6-tetra-O-acetyl-β-glucopyranosyl) flavone* (**10**)*.* Mp 222–223 °C; IR (KBr) υ_max_: 3442, 2961, 2917, 2847, 1758, 1621, 1572, 1501, 1457, 1235, 1211, 1071 cm^−1^; ^1^H NMR (CDCl_3_, 300.13 MHz) δ: 9.62 (3-OH), 8.24-8.18 (3H, m, H-5,2′,6′), 7.57–7.48 (3H, m, H-3′,4′,5′), 7.13 (1H, d, *J =* 2.2 Hz, H-8), 7.06 (1H, dd, *J =* 2.3 and 8.9 Hz, H-6), 5.36–5.16 (4H, m, H-1″,2″,3″,4″), 4.33–4.20 (2H, m, H6″a,b), 4.03–3.97 (1H, m, H-5″), 2.09 (3H, s, COCH_3_), 2.08 (3H, s, COCH_3_), 2.07 (3H, s, COCH_3_), 2.06 (3H, s, COCH_3_) ppm; ^13^C NMR (CDCl_3_, 75.47 MHz) *δ*: 172.9 (C-4), 170.6 (COCH_3_), 170.3 (COCH_3_), 169.6 (COCH_3_), 169.4 (COCH_3_), 160.9 (C-7), 156.8 (C-9), 138.4 (C-3), 131.1 (C-1′), 130.4 (C-4′), 128.8 (C-3′,5′), 127.7 (C-2′,6′), 127.4 (C-5), 116.6 (C-10), 115.4 (C-6), 104.3 (C-8), 98.4 (C-1″), 72.6 (C-3″), 72.6 (C-5″), 71.1 (C-2″), 68.3 (C-4″), 62.1 (C-6″), 20.8 (COCH_3_), 20.8 (COCH_3_), 20.7 (COCH_3_), 20.7 (COCH_3_) ppm; HRMS (ESI^+^) *m*/*z* calcd for C_29_H_28_O_13_ [M + H] 585.16027, found 585.15875.

### 3.3. Tumor Cell Growth Assay and Selectivity Index

The human tumor cell lines A375-C5 (melanoma), MCF-7 (breast adenocarcinoma), and NCI-H460 (non-small cell lung cancer) were grown in culture medium RPMI-1640 (Biochrom, Berlin, Germany), U251 (glioblastoma astrocytoma), U373 (glioblastoma astrocytoma), and U87MG (glioblastoma astrocytoma) were grown in culture medium DMEM (Biochrom), both supplemented with 5% heat-inactivated fetal bovine serum (FBS, Biochrom). All cell lines were maintained at 37 °C in a 5% CO_2_ humidified atmosphere (Hera Cell, Heraeus). Cell viability was routinely determined with Trypan Blue (Sigma-Aldrich) exclusion assay and all experiments were performed with exponentially growing cells, showing more than 95% viability.

The effect of compounds on the cell growth was evaluated according to the procedure adopted by the National Cancer Institute (NCI) in the ‘In vitro Anticancer Drug Discovery Screen’, which uses the protein-binding dye sulforhodamine B (SRB) to assess cell growth. Cells were plated in 96-well plates (0.05 × 106 cells/well) in complete culture medium and incubated at 37 °C. Twenty-four hours later, cells were treated with two-fold serial dilutions of the test compounds, ranging from 0 to 150 µM, for 48 h. Control groups received the same amount of sterile Dimethyl Sulfoxide (DMSO, Sigma-Aldrich), used as compounds solvent, up to 0.25% concentration. Then, cells were fixed in situ with 50% (*m*/*v*) trichloroacetic acid (Merck Millipore, Darmstadt, Germany), washed with distilled water and stained with Sulforhodamine B (SRB; Sigma-Aldrich) for 30 min at room temperature. The SRB-stained cells were washed 5 times with 1% (*v*/*v*) acetic acid (Merck Millipore) and left to dry at room temperature. SRB complexes were solubilized, by adding 10 mM Tris buffer (Sigma-Aldrich) for 30 min. Absorbance was measured at 515 nm in a microplate reader (Biotek Synergy 2,BioTek Instruments, Inc., Winooski, VT, USA). A dose–response curve was obtained for each cell line with each test compound, and the concentration that caused cell growth inhibition of 50% (GI_50_) was determined.

The degree of selectivity of the compounds was assessed by determining the selectivity index (SI) as the ratio of GI_50_ in non-tumor Human Pulmonary Alveolar Epithelial Cells (HPAEpiC) over GI_50_ in cancer cells. An SI value less than 2.0 indicates the general toxicity of the compound [44].

## 4. Conclusions

In this work, four acetylated flavonosides (**5**, **6**, **9**, and **10**), and one xanthonoside (**7**) were synthesized. As far as we know, compounds **9** and **10** are described for the first time. Compound **5** was synthesized without pyridine in a shorter reaction time. Applying MW irradiation to the synthesis of compounds **6** and **7** allowed to shorten the reaction times.

The in vitro growth inhibitory activity of compounds **5**–**7** and compounds **9** and **10** was investigated. 3-Hydroxy-7-(2,3,4,6-tetra-*O*-acetyl-β-glucopyranosyl) flavone (**10**) was the most active compound in all the human tumor cell lines tested with notable selectivity for cancer cells. The mechanism of action of this promising compound deserves to be further explored. The position of the sugar moiety seems to influence the activity of these compounds.

These results are a starting point from which to investigate more stable chemical modifications in order to optimize such molecules and develop detailed structure-activity relationship studies in future.

## Figures and Tables

**Table 1:** Cell growth inhibitory activity displayed by compounds **1**–**7**, **9**, and **10** and their calculated iLogP values *.

Compounds	GI_50_(μM)						iLogP
	A375-C5	MCF-7	NCI-H460	U251	U373	U87-MG	
**1**	>150	>150	>150	ND	ND	ND	2.43
**2**	>150	>150	>150	ND	ND	ND	2.03
**3**	>150	>150	>150	ND	ND	ND	1.42
**4**	10.81 ± 1.42	12.55 ± 5.23	12.80 ± 1.83	9.89 ± 0.53	10.24 ± 1.01	18.87 ± 1.36	2.01
**5**	9.65 ± 1.92	20.49 ± 2.14	14.32 ± 4.60	24.46 ± 4.08	20.17 ± 1.92	14.28 ± 1.62	5.44
**6**	>150	>150	>150	ND	ND	ND	4.03
**7**	58.06 ± 2.74	88.49 ± 0.72	99.90 ± 5.81	>150	>150	>150	3.08
**9**	10.44 ± 0.72	>150	>150	32.86 ± 0.042	109.16 ± 1.19	100.37 ± 9.61	4.35
**10**	7.34 ± 0.93	2.67 ± 0.49	7.61 ± 0.55	5.41 ± 0.83	6.42 ± 2.01	6.48 ± 0.67	3.55
Doxorubicin	0.014 ± 0.002	0.009 ± 0.001	0.009 ± 0.002	0.011 ± 0.004	0.009 ± 0.001	0.010 ± 0.002	-

Doxorubicin was used as positive control. GI_50_—concentration for 50% of maximal inhibition of cell proliferation. ND—not determined. * iLogP from SwissADME software using an in-house physics-based method implemented from [40].

**Table 2:** Selectivity index of the compounds **4**, **5**, **7**, **9**, **10***,* and control (Doxorubicin).

Compounds	HPAEpiC (GI_50_, µM)	Selectivity Index					
		A375-C5	MCF-7	NCI-H460	U251	U373	U87-MG
**4**	16.03 ± 1.38	1.48	1.28	1.25	1.62	1.57	0.85
**5**	14.13 ± 5.06	1.46	0.69	0.99	0.58	0.70	0.99
**7**	110 ± 7.07	1.89	1.24	1.10	-	-	-
**9**	91.40 ± 0.85	8.75			2.78	0.84	0.91
**10**	31.71 ± 1.63	4.32	11.88	4.17	5.68	4.94	4.89
Doxorubicin	0.004 ± 0.09	0.29	0.44	0.44	0.36	0.44	0.40

## Supplementary Materials

The following are available online, Figure S1. ^1^H and ^13^C NMR of compound **9**, Figure S2. ^1^H and ^13^C NMR of compound **10**, Figure S3. HRMS for compound **9**, Figure S4. HRMS for compound **10**.
