# Supplementary material for: Synthesis of New Glycosylated Flavonoids with Inhibitory Activity on Cell Growth
Source: Molecules. 2018 May 5;23(5):1093. doi: 10.3390/molecules23051093 (PMC6102538; doi:10.3390/molecules23051093)
Supplement: Supplementary file 1 [file molecules-23-01093-s001.pdf]

SUPPORTING INFORMATION

Synthesis of New Glycosylated Flavonoids with Inhibitory Activity on Cell Growth

Ana R. Neves [1,2,†], Marta Correia-da-Silva [1,2,†], Patrícia M. A. Silva [3], Diana Ribeiro [3], Emília Sousa [1,2,*], Hassan Bousbaa [2,3] and Madalena Pinto [1,2]

1 Laboratory of Organic and Pharmaceutical Chemistry, Department of Chemical Sciences, Faculty of Pharmacy, University of Porto, Rua Jorge Viterbo Ferreira, 228, 4050-313 Porto, Portugal; anarcneves92@gmail.com (A.R.N.); m_correiadasilva@ff.up.pt (M.C.-d.-S.); madalena@ff.up.pt (M.P.)

2 Interdisciplinary Centre of Marine and Environmental Research (CIIMAR), University of Porto, Terminal de Cruzeiros do Porto de Leixões Avenida General Norton de Matos P 4450-208 Matosinhos, Portugal; hassan.bousbaa@iucs.cespu.pt

3 CESPU, Institute of Research and Advanced Training in Health Sciences and Technologies (IINFACTS), Rua Central de Gandra, 1317, 4585-116 Gandra, Portugal; patricia_masilva@hotmail.com (P.M.A.S.); dianaroberta@ua.pt (D.R.)

* Correspondence: esousa@ff.up.pt; Tel.: +351-220428689

† These authors contributed equally to this work.

Table of Contents

NMR spectra 2

Figure S1. ^{1}H and ^{13}C NMR of compound 9 2

Figure S2. ^{1}H and ^{13}C NMR of compound 10 3

HRMS spectra 4

Figure S3. HRMS for compound 9 4

Figure S4. HRMS for compound 10 5

NMR spectra

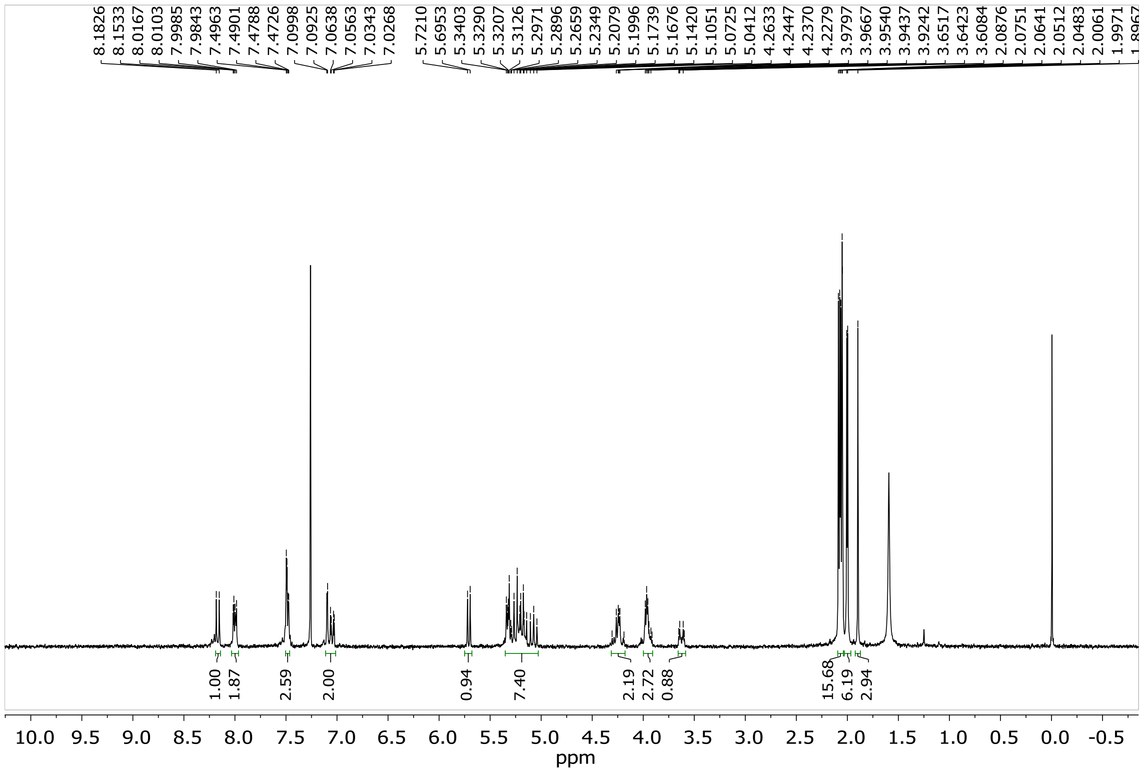

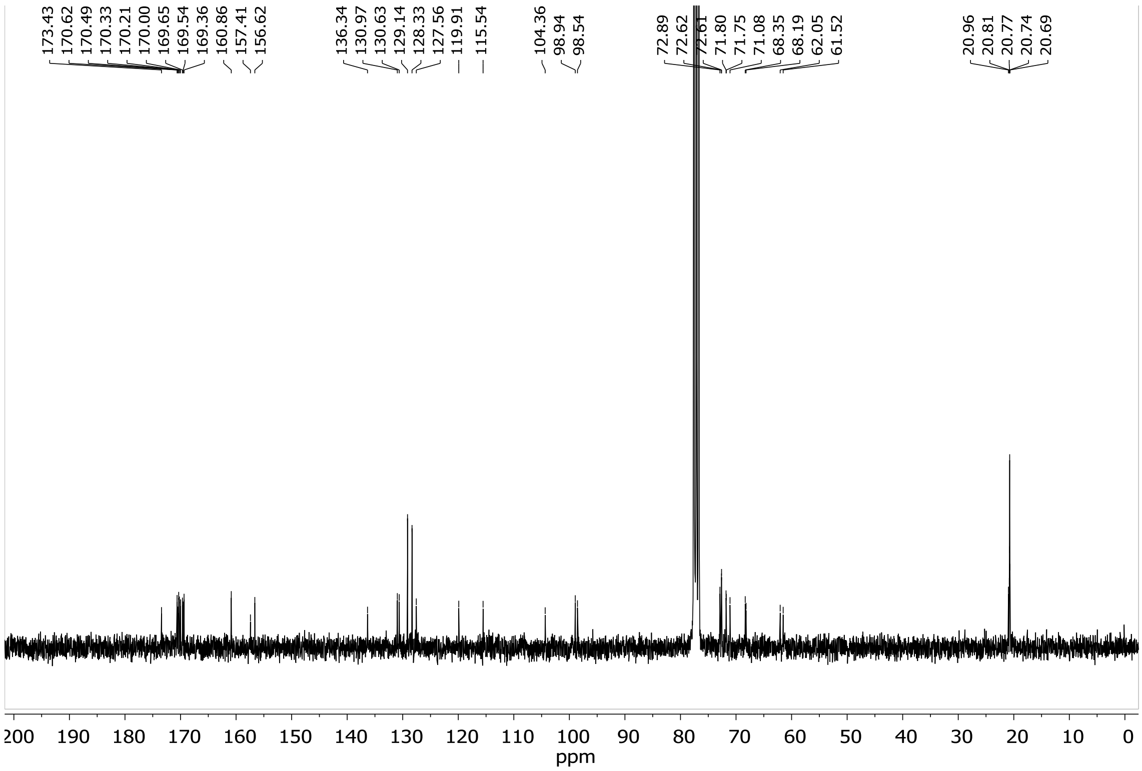

Figure S1. ^{1}H and ^{13}C NMR of compound 9

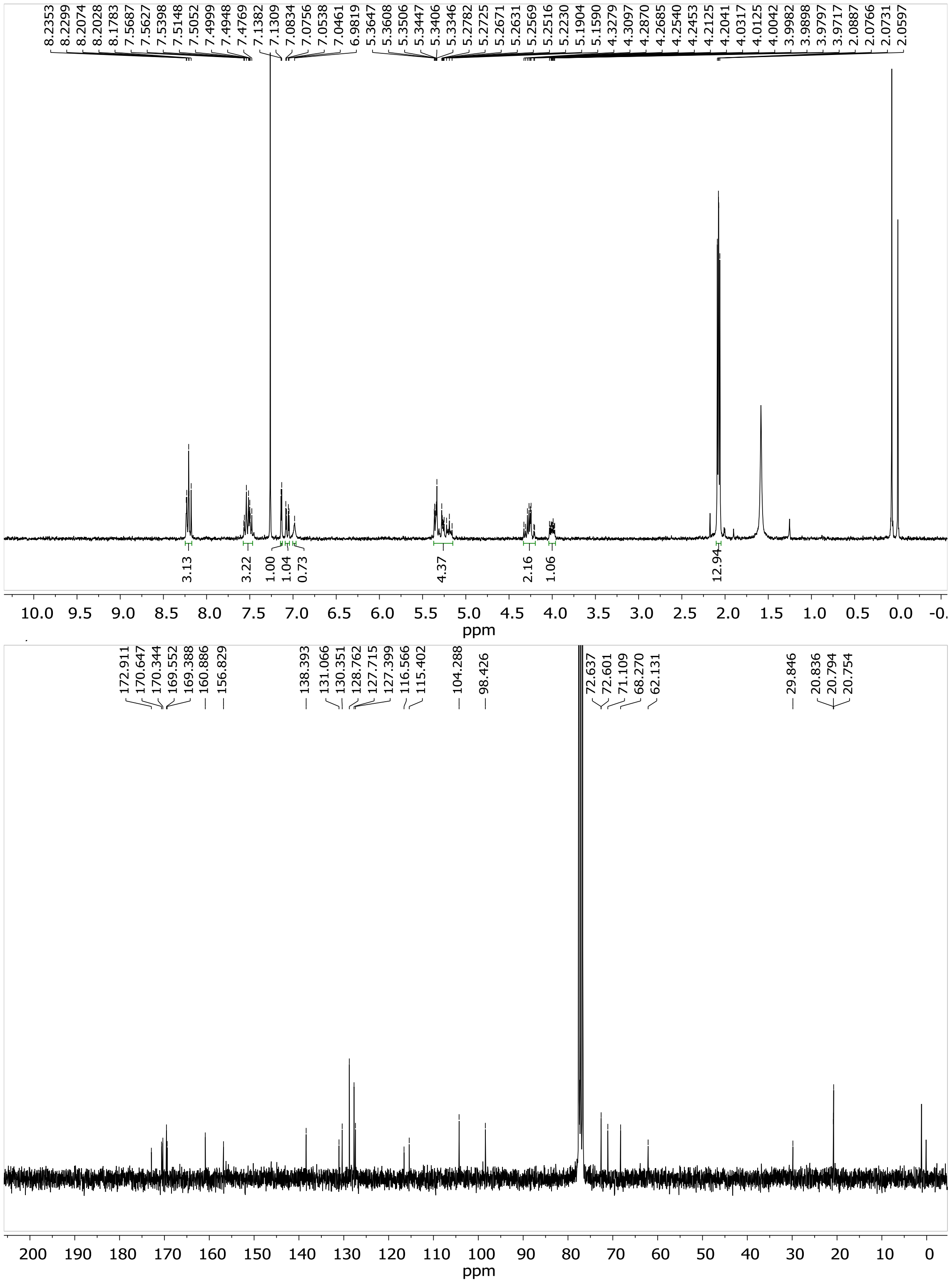

Figure S2. ^{1}H and ^{13}C NMR of compound 10

HRMS spectra

Mass Spectrum Molecular Formula Report

Analysis Info **Electrospray (ESI)**

Analysis Name	D:\Data\AEMPRESAS\JRMP1410291_000001.d	Acquisition Date	5/12/2014 12:39:56 PM
Sample Name	F3-F7_AC	Instrument	apex-Qe
Comment			

Acquisition Parameter

Capillary Exit 300.0 V

Skimmer 1 20.0 V

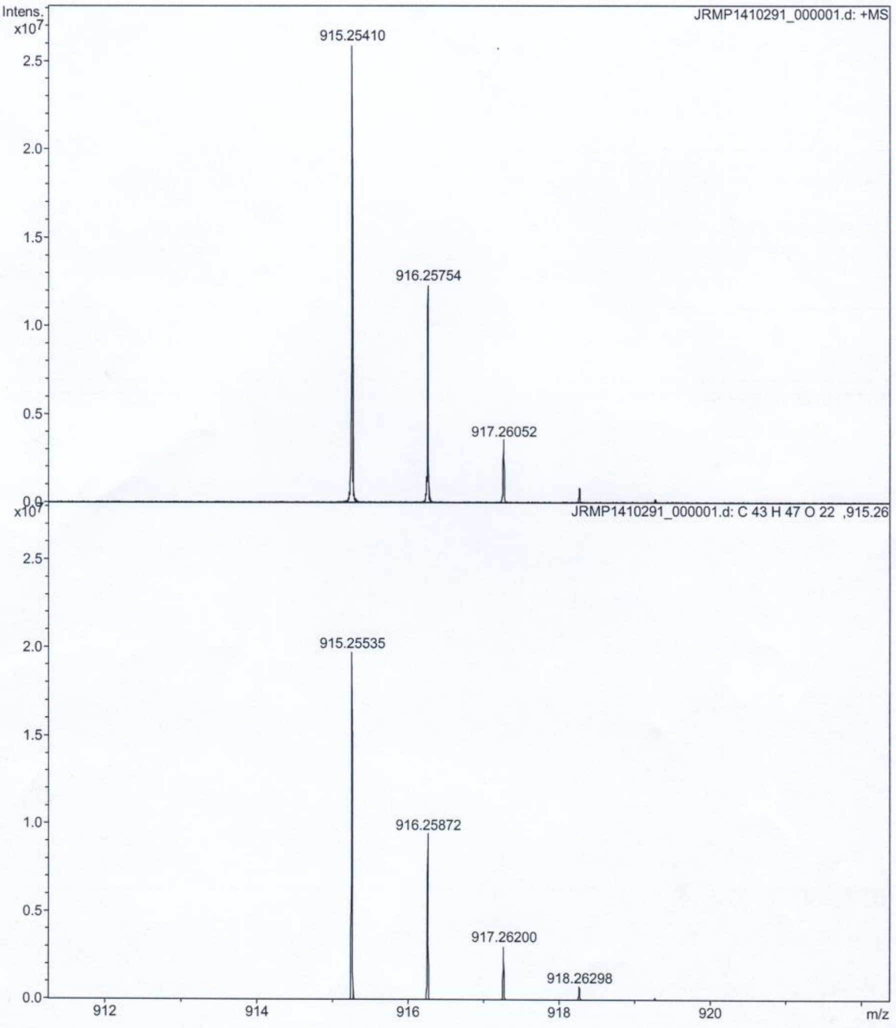

U. Vigo/CACTI/DEyP/APEX-Qe

Page 1 of 2

Mass Spectrum Molecular Formula Report

Meas. m/z	#	Formula	Score	m/z	err [mDa]	err [ppm]	mSigma	rdb	e⁻ Conf	N-Rule
915.25410	1	C 43 H 47 O 22	100.00	915.25535	1.25	1.36	19.4	20.5	even	ok

Figure S3. HRMS for compound 9

Mass Spectrum Molecular Formula Report

Analysis Info **Electrospray (ESI)**

Analysis Name D:\Data\AUVIGO\JRMP17028716_000001.d

Sample Name 3.7FMonoGluAc

Comment

Acquisition Date 4/27/2017 4:15:25 PM

Instrument apex-Qe

Acquisition Parameter

Capillary Exit 300.0 V

Skimmer 1 20.0 V

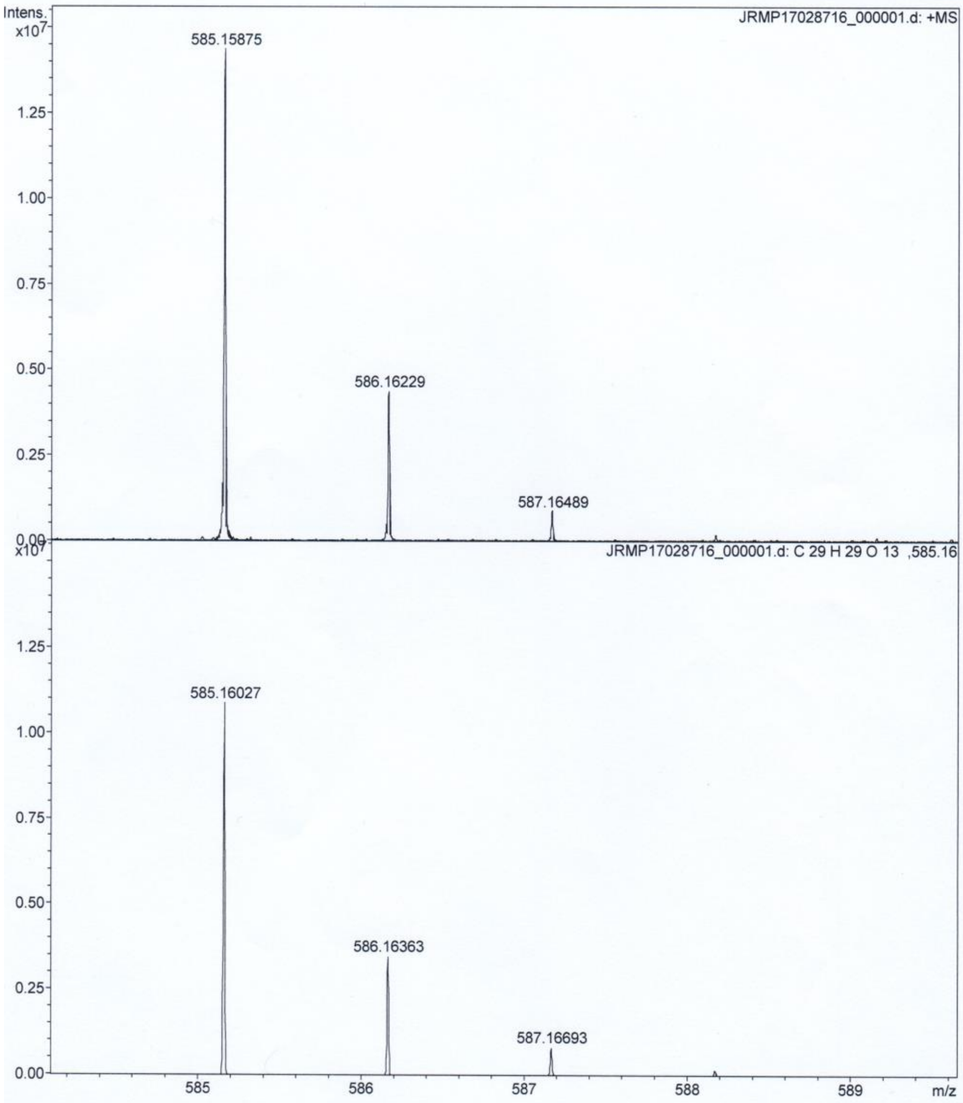

U. Vigo/CACTI/DEyP/APEX-Qe

Mass Spectrum Molecular Formula Report

Meas. m/z	#	Formula	Score	m/z	err [mDa]	err [ppm]	mSigma	rdb	e⁻ Conf	N-Rule
585.15875	1	C 29 H 29 O 13	100.00	585.16027	1.52	2.59	11.4	15.5	even	ok
	2	C 11 H 37 O 26	7.29	585.15676	-1.99	-3.41	86.3	-6.5	even	ok

Figure S4. HRMS for compound 10
